# Supplementary material for: Evaluation of Antimicrobial Activity by Marine Nocardiopsis dassonvillei against Foodborne Listeria monocytogenes and Shiga Toxin-Producing Escherichia coli
Source: Microorganisms. 2023 Oct 12;11(10):2539. doi: 10.3390/microorganisms11102539 (PMC10609338; doi:10.3390/microorganisms11102539)
Supplement: Supplementary file 1 [file microorganisms-11-02539-s001.zip › microorganisms-2577056-supplementary.pdf]

microorganisms

Supplementary data

Evaluation of antimicrobial activity by marine *Nocardiopsis dassonvillei* against foodborne *Listeria monocytogenes* and Shiga toxin-producing *Escherichia coli*

Siyanda S. Ngema[1], Solomuzi H. Khumalo[1], Michael C. Ojo[1], Ofentse J. Pooe[2], Tsolanku S. Malilehe[3], Albert K. Basson[1] and Evelyn Madoroba[1*]

[1] Department of Biochemistry and Microbiology; ngemasiyanda05@gmail.com; elsherawy7@gmail.com; mikekonyegwachie2015@gmail.com; BassonA@unizulu.ac.za; evelyn.madoroba@gmail.com; MadorobaE@unizulu.ac.za
[2] Discipline of Biochemistry, School of Life Sciences, University of KwaZulu-Natal, Durban, South Africa PooeO@ukzn.ac.za
[3] Department of Water and Sanitation, University of Limpopo, Private Bag X1106, Polokwane, 0727, South Africa tsolanku.maliehe@ul.ac.za
* Correspondence: evelyn.madoroba@gmail.com; MadorobaE@unizulu.ac.za

Citation: To be added by editorial staff during production.

Academic Editor: Firstname Lastname

Received: date
Accepted: date
Published: date

Publisher's Note: MDPI stays neutral with regard to jurisdictional claims in published maps and institutional affiliations.

Copyright: © 2022 by the authors. Submitted for possible open access publication under the terms and conditions of the Creative Commons Attribution (CC BY) license (https://creativecommons.org/licenses/by/4.0/).

Table S1. Compounds identified by GC-MS in the chloroform extract of *N. dassonvillei* SOD(B)ST2SA2

Peak#	R.Time	Area	Area%	Height	Height%	Name
1	9.130	211980	0.23	65206	0.28	Acetamide, N-(.beta.-mercaptoethyl)-
2	9.175	373287	0.40	86963	0.38	1,2,4,5-Tetrazine, hexahydro-1,2,4,5-tetramethy
3	9.250	206326	0.22	76562	0.33	2-Heptanone, 5-methyl-
4	9.300	35784	0.04	64479	0.28	Cyclohexan-1,4,5-triol-3-one-1-carboxylic acid
5	9.315	146328	0.16	64669	0.28	Butanamine, 2,2-dinitro-N-methyl-
6	9.380	139656	0.15	60365	0.26	(3s)-Pentanol, 4,4-dimethyl-(2s)-[(tert.butyloxy
7	9.430	307005	0.33	50129	0.22	dl-3-Aminoisobutyric acid, N-methyl-, methyl e
8	10.110	175018	0.19	70704	0.31	2,4(1H,3H)-Pyrimidinedione, dihydro-5-hydrox
9	10.155	226458	0.25	86358	0.38	Tetraacetyl-d-xylonic nitrile
10	10.190	225361	0.24	105667	0.46	2-Acetylamino-3-hydroxy-propionic acid
11	10.220	267666	0.29	132157	0.58	Ethylamine, 2-(adamantan-1-yl)-1-methyl-
12	10.260	123177	0.13	147519	0.64	Acetamide, 2,2,2-trichloro-
13	10.320	884208	0.96	211519	0.92	n-Hexadecanoic acid
14	10.365	229977	0.25	197549	0.86	3-Hexene-2,5-diol
15	10.395	377728	0.41	243640	1.06	(+-)-2-Hydroxyoctanoic acid, acetate
16	10.410	683729	0.74	262216	1.14	Propanedioic acid, propyl-
17	10.475	991521	1.07	314815	1.37	Acetic acid, [(aminocarbonyl)amino]oxo-
18	10.523	1462876	1.58	347052	1.51	Acetic acid, mercapto-, hexyl ester
19	10.580	381422	0.41	337616	1.47	2-Amino-1,3-propanediol
20	10.600	2008032	2.17	326741	1.42	3-Butenamide
21	10.745	496823	0.54	254460	1.11	Pterin-6-carboxylic acid
22	10.780	487622	0.53	234248	1.02	Dec-9-en-6-oxo-1-ylamide
23	10.795	1216371	1.32	240812	1.05	Tetraacetyl-d-xylonic nitrile
24	11.396	183148	0.20	68991	0.30	Carbonochloridic acid, decyl ester
25	12.263	363340	0.39	197357	0.86	2,5-Cyclohexadiene-1,4-dione, 2,6-bis(1,1-dim
26	12.672	347074	0.38	79378	0.35	1b,4a-Epoxy-2H-cyclopenta[3,4]cyclopropa[8,
27	12.793	537547	0.58	197146	0.86	Phenol, 2,4-bis(1,1-dimethylethyl)-
28	12.840	141944	0.15	66831	0.29	Methyl 4,6-di-O-acetyl-2,3-diacetamido-2,3-di
29	12.880	148018	0.16	56144	0.24	4H-1,3,4-Triazol-3-amine, N-dimethylaminome
30	13.505	355849	0.39	183223	0.80	1-Pentadecene
31	13.575	3170477	3.43	1224825	5.33	Diethyl Phthalate
32	13.700	259201	0.28	126006	0.55	4,5-Dicarboxy-1,2,3-triazole
33	13.731	641870	0.69	128624	0.56	2H-Azepine-2-thione, hexahydro-
34	14.543	1021053	1.10	209437	0.91	1H-Pyrrolo[2,3-b]pyridine-2,6-dione, 3,3a,4,5-
35	14.650	93480	0.10	73437	0.32	Cyclopentanecarboxylic acid, 2-acetyl-5-methy
36	14.701	357455	0.39	126085	0.55	Acetamide, N-(2,4-dimethylphenyl)-
37	14.811	771031	0.83	296529	1.29	Phenol, 4-(1,1,3,3-tetramethylbutyl)-
38	14.886	1046732	1.13	336573	1.47	1-(2,6-Dimethyl-4-propoxyphenyl)propan-1-on
39	14.936	446275	0.48	229748	1.00	N-(Chroman-5-yl)acetamide
40	15.003	1315900	1.42	296823	1.29	1,3-Cyclopentadiene, 2,3,4,5-tetramethyl-1-(4-
41	15.110	393725	0.43	193558	0.84	Benzestrol
42	15.135	285726	0.31	114745	0.50	Phenol, 3,5-diethyl-
43	15.214	769338	0.83	189619	0.83	Phenol, 2-methyl-4-(1,1,3,3-tetramethylbutyl)-
44	15.343	1613034	1.75	475671	2.07	Psicofuranine
45	15.410	896182	0.97	196814	0.86	Phenol, 2-methyl-4-(1,1,3,3-tetramethylbutyl)-
46	15.510	174144	0.19	78477	0.34	Pentadec-7-ene, 7-bromomethyl-
47	15.577	2188129	2.37	1099135	4.79	1-Octadecene
48	15.650	265908	0.29	121604	0.53	Decane, 2,3,5,8-tetramethyl-
49	15.952	508714	0.55	185490	0.81	Isopropyl myristate
50	16.134	102837	0.11	54362	0.24	9,9-Dimethoxybicyclo[3.3.1]nona-2,4-dione
51	16.237	427694	0.46	200054	0.87	2-Pentadecanone, 6,10,14-trimethyl-
52	16.577	90503	0.10	51769	0.23	1,2-Benzenedicarboxylic acid, bis(2-methylpro
53	16.792	426637	0.46	112113	0.49	Pyrrolo[1,2-a]pyrazine-1,4-dione, hexahydro-3
54	16.965	495206	0.54	63850	0.28	Bromoacetic acid, tridecyl ester
55	17.366	1893030	2.05	733144	3.19	7,9-Di-tert-butyl-1-oxaspiro(4,5)deca-6,9-diene
56	17.519	724160	0.78	193773	0.84	Hexadecanoic acid, methyl ester
57	17.675	1600267	1.73	546368	2.38	Benzenepropanoic acid, 3,5-bis(1,1-dimethylet
58	17.779	338430	0.37	76462	0.33	2-Dodecen-1-yl(-)succinic anhydride
59	17.981	338870	0.37	86571	0.38	Glutaric acid, dodecyl 2-methoxyphenyl ester

Table S1 continued

Peak#	R.Time	Area	Area%	Height	Height%	Name
60	18.196	1281602	1.39	423387	1.84	Dibutyl phthalate
61	18.399	5411656	5.86	1144550	4.98	Pentadecanoic acid
62	18.745	693785	0.75	170359	0.74	5-Eicosene, (E)-
63	18.861	8517166	9.22	2610749	11.37	1-Octadecene
64	18.985	559030	0.60	165914	0.72	Hexadecane, 4-methyl-
65	19.151	266106	0.29	63250	0.28	1H-1,2,4-Triazole, 1-octadecanoyl-
66	19.450	451454	0.49	155868	0.68	2H-2,4a-Ethanonaphthalen-8(5H)-one, hexahyd
67	19.495	888029	0.96	223424	0.97	Isopropyl palmitate
68	19.997	237839	0.26	74242	0.32	Eicosanoic acid
69	20.352	296490	0.32	63663	0.28	Pentadecanoic acid
70	20.774	210326	0.23	50097	0.22	18,19-Secoyohimban-19-oic acid, 16,17,20,21-
71	21.450	480308	0.52	92181	0.40	trans-2-Dodecen-1-ol, pentafluoropropionate
72	21.475	100914	0.11	73016	0.32	2-(2-Carbamoylethyl)-2,3-dihydro-6-hydroxy-3
73	21.647	690627	0.75	126637	0.55	10-Octadecenoic acid, methyl ester
74	22.096	823511	0.89	138658	0.60	Phytol, acetate
75	23.398	19351394	20.94	2126400	9.26	6-Octadecenoic acid, (Z)-
76	24.182	1642259	1.78	282620	1.23	Octadecanoic acid, 2-(2-hydroxyethoxy)ethyl e
77	24.780	1049722	1.14	181487	0.79	Tetradecanamide
78	24.875	96115	0.10	86818	0.38	Cyclohexanol, 2-methyl-, cis-
79	24.909	643379	0.70	108932	0.47	Decane, 4-cyclohexyl-
80	25.151	7228918	7.82	1165658	5.08	Trifluoroacetoxy hexadecane
81	25.376	626731	0.68	110530	0.48	2-methyltetracosane
82	25.796	597973	0.65	92932	0.40	Tetracosyl acetate
83	27.959	1366662	1.48	209523	0.91	N,N-Dimethyldodecanamide
84	30.513	2474954	2.68	316980	1.38	2-Propenoic acid, pentadecyl ester
85	30.645	47976	0.05	53719	0.23	2-Oxepanone, 7-butyl-

Ciprofloxacin

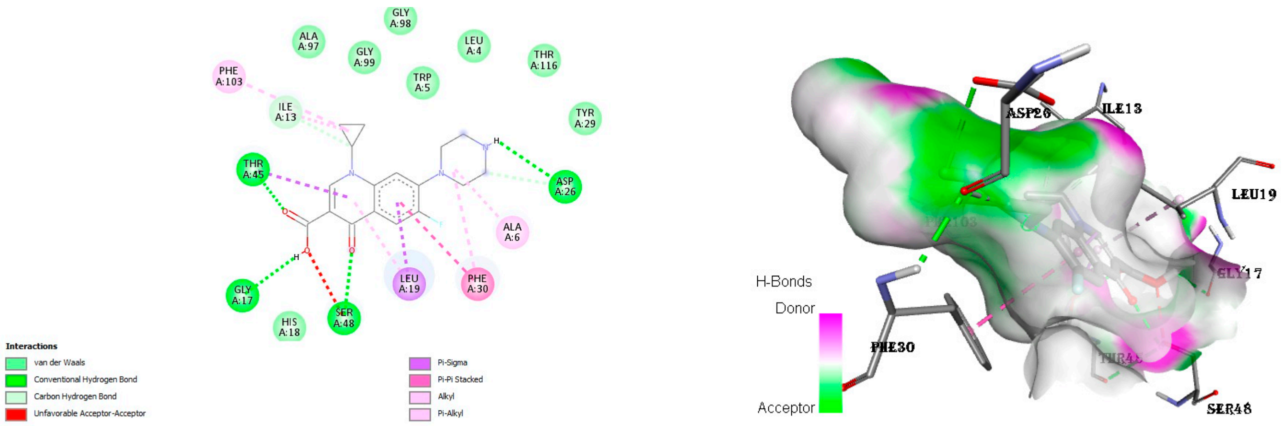

Figure S1. 2D and 3D binding interactions of ciprofloxacin against dihydrofolate reductase (PDB ID: 1DIS), respectively. The 3D interactions show the ligand in a binding pocket of the enzyme. Dashed lines indicate the interactions between the ligands and the amino acids of the enzyme.

Pentadecanoic acid

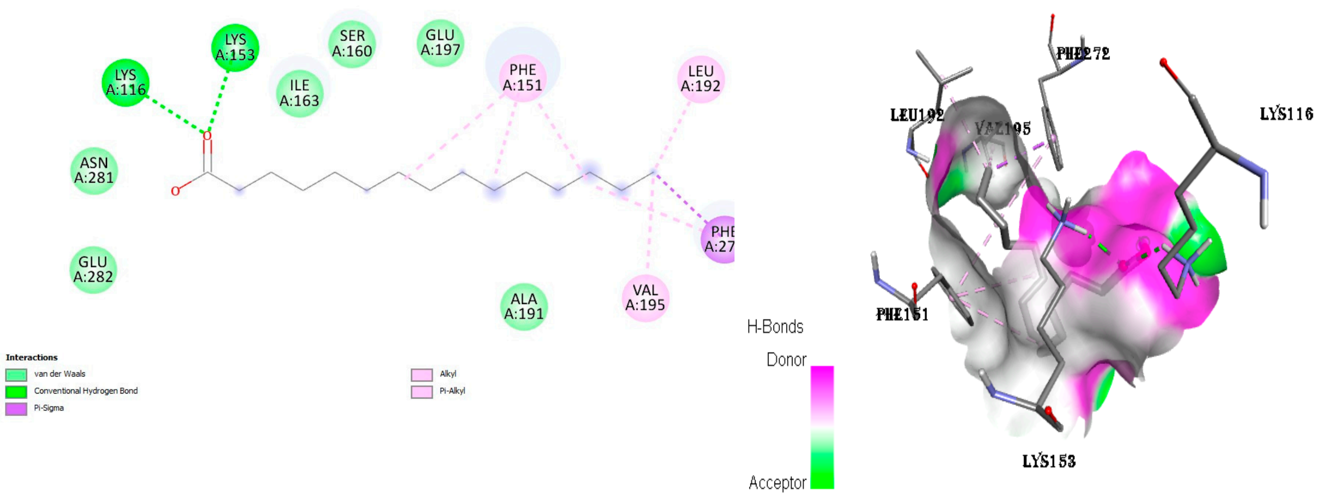

Diethyl phthalate

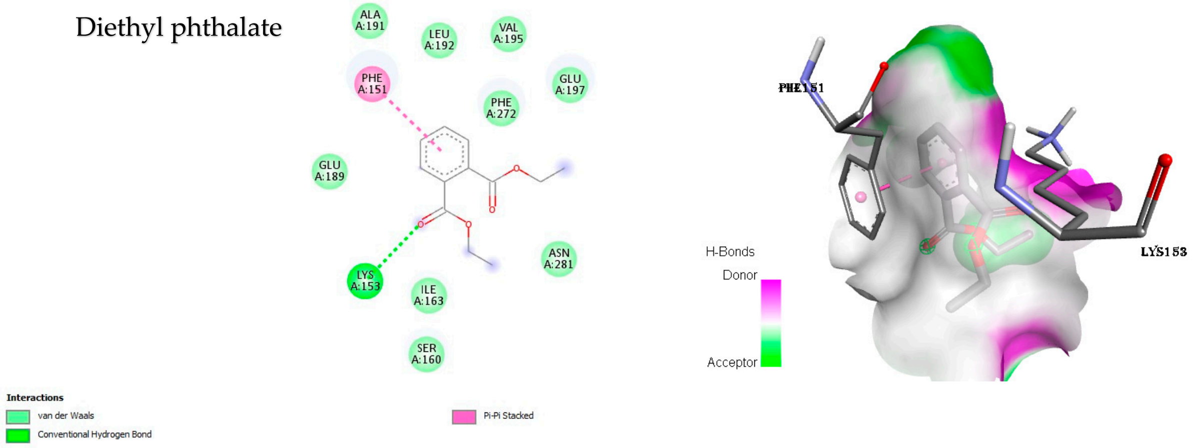

1-Octadecene

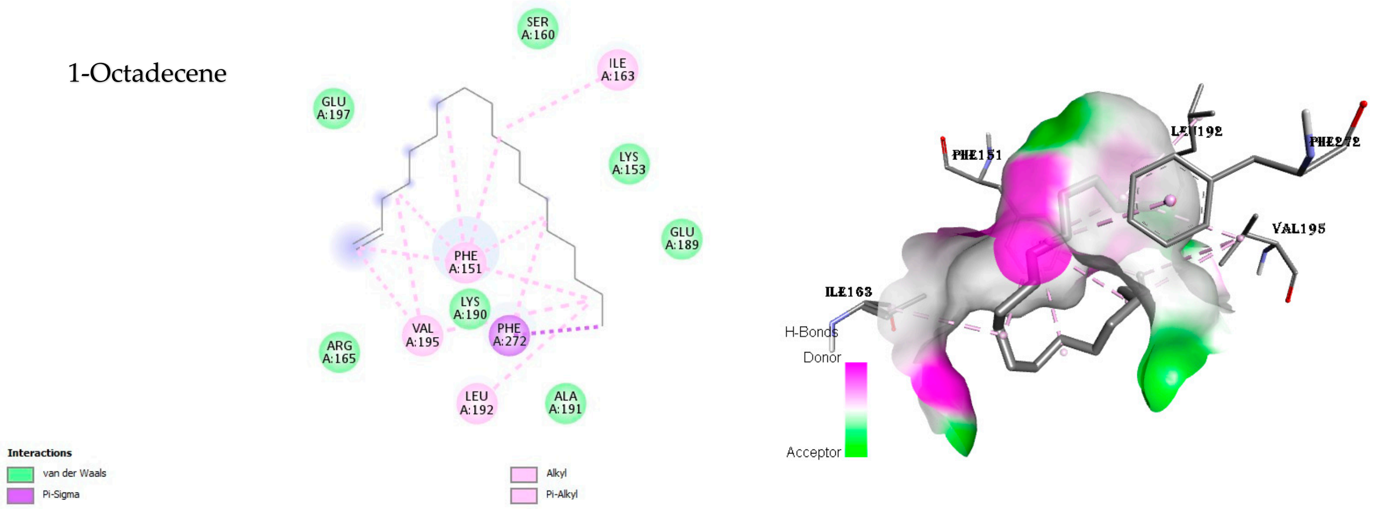

Figure S2. 2D and 3D binding interactions of compounds against D-alanine:D-alanine ligase (DDl) (PDB ID: 2Zdg), respectively. The 3D interactions show the ligand in a binding pocket of the enzyme. Dashed lines indicate the interactions between the ligands and the amino acids of the enzyme.

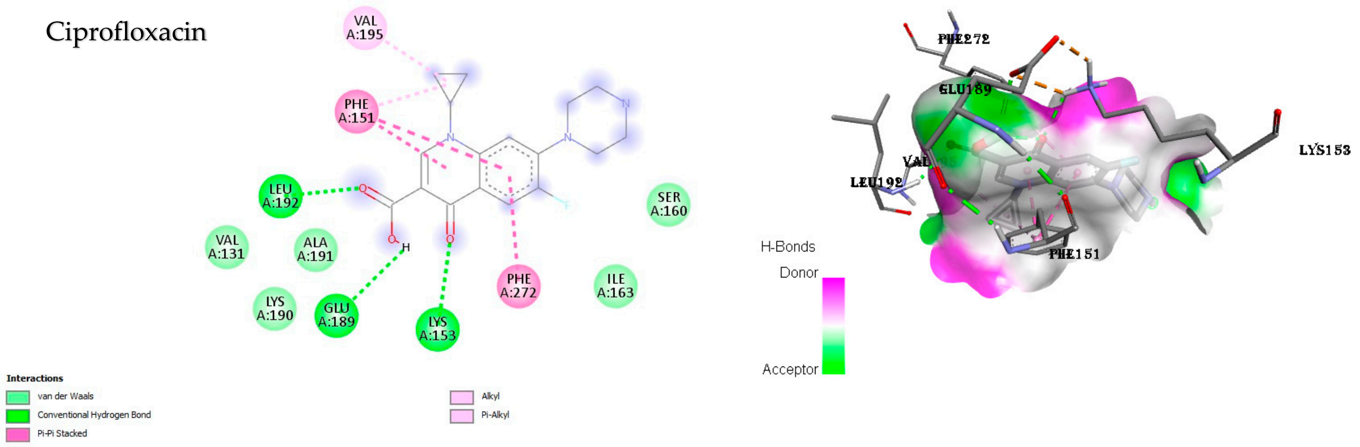

Figure S3. 2D and 3D binding interactions of ciprofloxacin against DDl (PDB ID: 2Zdg), respectively. The 3D interactions show the ligand in a binding pocket of the enzyme. Dashed lines indicate the interactions between the ligand and the amino acids of the enzyme.

Pentadecanoic acid

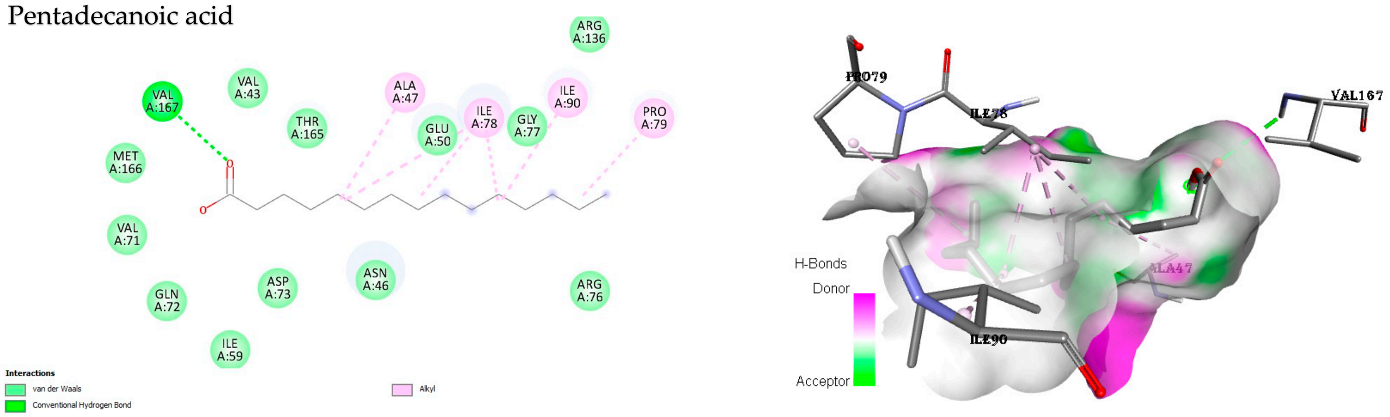

Diethyl phthalate

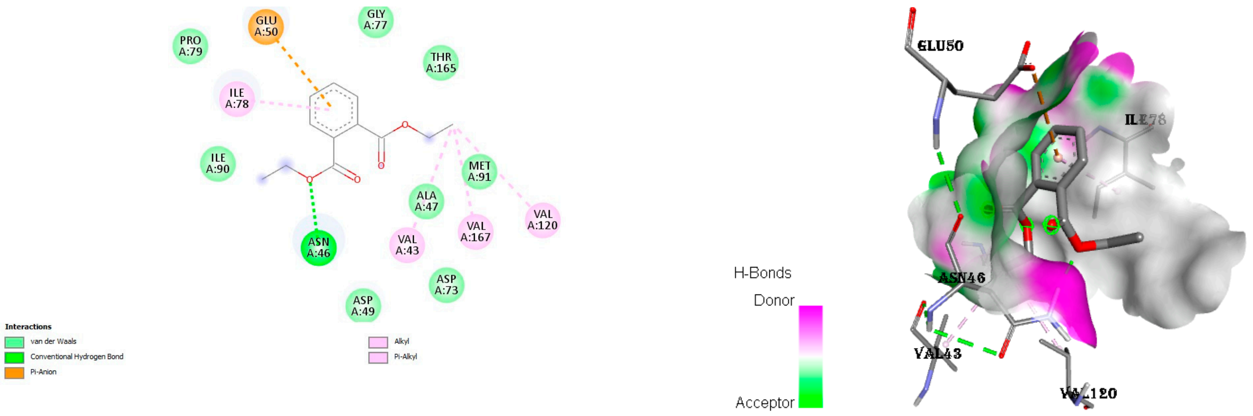

1-Octadecene

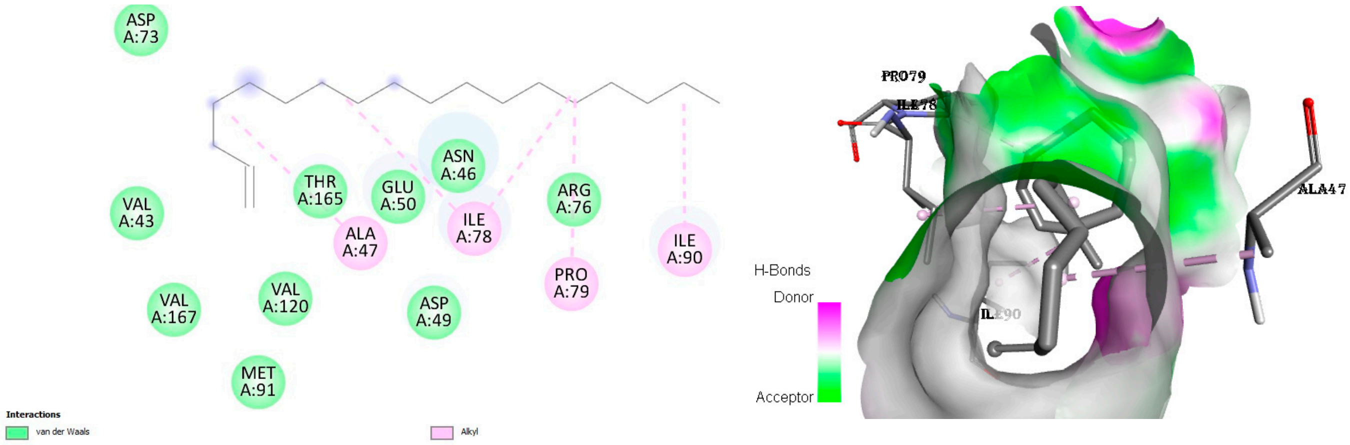

Figure S4. 2D and 3D binding interactions of compounds against DNA gyrase B (PDB ID: 1KZN), respectively. The 3D interactions show the ligand in a binding pocket of the enzyme. Dashed lines indicate the interactions between the ligands and the amino acids of the enzyme.

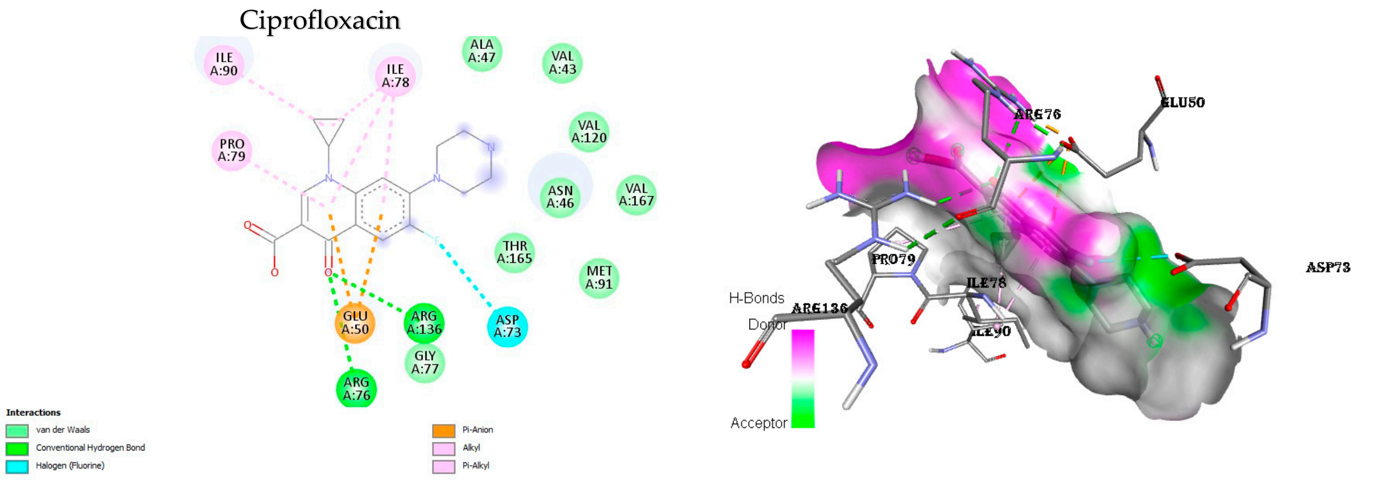

Figure S5. 2D and 3D binding interactions of ciprofloxacin against DNA gyrase B (PDB ID: 1KZN), respectively. The 3D interactions show the ligand in a binding pocket of the enzyme. Dashed lines indicate the interactions between the ligand and the amino acids of the enzyme.

Sequence of *Nocardiopsis dassonvillei* strain SOD(B)ST2SA2 that showed activity during secondary screening for antibacterial activity

>SOD-B_907-R

CTCCCCSGGCSGGGGGCGCTTATGCGTTAGCTACGGCGCGGAAACCGTGGAAAGTCCCCACACCTAKYGCCC
AACGTTTACGGCATGGACTACCAGGGTATCTAATCCTGTTCGCTCCCCATGCTTTCGCTCCTCAGCGTCAGGTA
AGGCCCAGAGACCCGCCTTCGCCACCGGTGTTCCTCCTGATATCTGCGCATTTCACCGCTACACCAGGAATTC
CAGTCTCCCCTACCTACCTCTAGCATGCCCGTATCCACTGCAGAACCGGAGTTAAGCCCCGGTCTTTCACAGC
AGACGCGACACGCCGCCTACGAGCTCTTTACGCCCAATAATTCCGGACAACGCTCGGACCCTACGTATTACC
GCGGCTGCTGGCACGTAGTTAGCCGGTCCTTATTCCCCACCTACCGTCAACCCGAAGAGAACTTCGAGCCTGC
GTTGGTGGTAAAAGAGGTTTACAACCCGAAGGCCGTCATCCCCCACGCGGCGTCGCTGCGTCAGGCTTTCGC
CCATTGCGCAATATTCCCCACTGCTGCCTCCCGCAGGAGTCTGGGCCGTGTCTCAGTCCCAGTGTGGCCGGTC
GCCCTCTCAGGCCGGCTACCCGTAATCGCCTTGGTAGGCCGTTACCCCACCAACAAGCTGATAGGCCGCGAG
CCCATCCCTGACCGAAAAACTTTCCACCCTCCACCATGAGGTGGCGGGTCGTATCCGGTATTAGACGGCGTTT
CCACCGCTTATCCCGGAGTCAGGGGCAGGTTGCTCACGTGTTACTCACCCGTTCGCCGCTCGTGTACCCCGAA
AGGGCCTTACCGCTCGACTTGCATGTGTTAAGCACGCCGCCAGCGTTCGTCCTGAGCCATAWWYWMAAMTY
CT

>SOD-B_1492-R

CMGGCCCCMMCCTTCACTCACTCCCTCCMACAAGGGGTTAGGCCG-
CAAGTTTCGGGTGTT-
GCCGACTTTCWTGACGTGACGGGCGGTGTGTACAAGGCCCGGGAACGTATTCACCGCGGC
GTTGCTGATCCGCGATTACTAGCGACTCCACCTTCATGGGGTCGAGTT-
GCAGACCCCAATCCGAACTGAGACCGGCTTTTAGGGATTCGCTCCACCTTACGGTATCGC
ACGCCCATTGTACCGGCCATTGTAGCATGTTTGCAGCCCAAGACATAAGGGG-
CATGATGACTTGACGTCATCCCCACCTTCCTCCGAGTTGACCCCGGCAGTCTCCCATGAGT
CCCCACCATTACGTGCTGGCAACATGGAACAAGGGTTGCGCTCGTTGCGG-
GACTTAACCCAACATCTCACGACACGAGCTGACGACAGCCATGCACCACCTGTCACCCGC
CAACTAAATGACCTCACATCTCTGCGAGTCCACGGGTGATGTCAAACCTTGG-
TAAGGTTCTTCGCGTTGCGTCGAATTAAGCAACATGCTCCGCCGCTTGTGCGGGCCCCCGT

CAATTCCTTTGAGTTTTAGCCTTGCGGCCG-
TACTCCCCAGGCGGGGCGCTTAATGCGTTAGC-
TACGGCGCGGAAACCGTGGAAAGTCCCCACACCTAGCGCCCAACGTTTACGGCATGGACT
ACCAGGG-
TATCTAATCCTGTTCGCTCCCCATGCTTTCGCTCCTCAGCGTCAGGTAAGGCCCAGAGACC
CGCCTTCGCCACCGGTGTTCCTCCTGATATCTGCGCATTTCACCGCTACACCAG-
GAATTCCAGTCTCCCCTACCTACCTCTAGCATGCCCGTATCCACTGCAGAACCGGAGTTAA
GCCCCGGTCTTTCACAGCAGACGCGACACGCCGCCTACGAGCTCTTTACGCCCA-
TAATTCCG-
GACAACGCTCGGACCCTACGTATTACCGCGGCTGCTGGCACGTAGTTAGCCGGTCCTTATT
CCCCACCTACCGTCAACCCGAAGARAACTTCGAGCCTGCGTTGGTGGTAAA-
GAGGTTTACAACCGAAGGCGTCATCCCCCACGCGCGTCGCTGCGTCAGCTTCGCATGGCC
AAWWTCCATGCTGCTCCGCAGAGCTGACGKGTYYCAGTCAAGKTGAC-
GTCGCCYTCAGCGATACGATGCATGTGCGTACTCACAMAGCKATAGCGCGGACCCATCT
